# Comparison of Single-Stranded DNA Probes Conjugated with Magnetic Particles for Trans-Cleavage in Cas12a-Based Biosensors

**DOI:** 10.3390/bios13070700

**Published:** 2023-07-01

**Authors:** Aleksandr V. Ivanov, Irina V. Safenkova, Anatoly V. Zherdev, Yi Wan, Boris B. Dzantiev

**Affiliations:** 1 A.N. Bach Institute of Biochemistry, Research Centre of Biotechnology of the Russian Academy of Sciences, 119071 Moscow, Russia; a.ivanov@fbras.ru (A.V.I.); zherdev@inbi.ras.ru (A.V.Z.); dzantiev@inbi.ras.ru (B.B.D.); 2 State Key Laboratory of Marine Resource Utilization in South China Sea, Marine College, Hainan University, Haikou 570228, China; 993602@hainanu.edu.cn

**Keywords:** CRISPR–Cas12, magnetic particles, trans-cleavage, DNA amplification, ssDNA probe, isothermal amplification

## Abstract

Biosensors based on endonuclease Cas12 provide high specificity in pathogen detection. Sensitive detection using Cas12-based assays can be achieved using trans-cleaved DNA probes attached to simply separated carriers, such as magnetic particles (MPs). The aim of this work was to compare polyA, polyC, and polyT single-stranded (ss) DNA with different lengths (from 10 to 145 nt) as trans-target probes were immobilized on streptavidin-covered MPs. Each ssDNA probe was labeled using fluorescein (5′) and biotin (3′). To compare the probes, we used guide RNAs that were programmed for the recognition of two bacterial pathogens: *Dickeya solani* (causing blackleg and soft rot) and Erwinia amylovora (causing fire blight). The Cas12 was activated by targeting double-stranded DNA fragments of *D. solani* or *E. amylovora* and cleaved the MP–ssDNA conjugates. The considered probes demonstrated basically different dependencies in terms of cleavage efficiency. PolyC was the most effective probe when compared to polyA or polyT probes of the same length. The minimal acceptable length for the cleavage follows the row: polyC < polyT < polyA. The efficiencies of polyC and polyT probes with optimal length were proven for the DNA targets’ detection of *D. solani* and *E. amylovora*. The regularities found can be used in Cas12a-based detection of viruses, bacteria, and other DNA/RNA-containing analytes.

## 1. Introduction

Biotechnologies based on clustered regularly interspaced short palindromic repeats (CRISPR)–CRISPR-associated protein (Cas) systems are being developed rapidly [1]. Cas12a is the most widely and successfully used enzyme in biosensors, able to detect double-stranded (ds)DNA targets [2,3]. Its functional complex comprises Cas12a and either CRISPR (cr)RNA or an artificial guide (g)RNA. Cas12 scans dsDNA to find a protospacer-adjacent motif (PAM). The gRNA recognizes and binds 20 nucleotides (nts) in the complementary chain of dsDNA (spacer). Then, Cas12a makes an asymmetric cut of both stands of the dsDNA, which is called cis-cleavage [4,5]. Following cis-cleavage, conformation changes in the active center of Cas12a, enabling cleavage of any single-stranded (ss)DNA longer than 5 nt. Such nonspecific activity is called trans-cleavage. Trans-cleavage causes multiple cuts and is 10 times faster than cis-cleavage [6,7]. Therefore, biosensing processes typically involve the recognition of target dsDNA using Cas12a–gRNA and the following trans-cleavage of the ssDNA reporter. The reporter can be labeled using detected fluorophore, a quencher, or an affine tag. The nucleic acid target could be preliminarily amplified to increase the detection sensitivity with an isothermal preamplification step (e.g., LAMP, RPA, NASBA, etc.) [8,9]. The bioanalytical applications of Cas12a were transformed into such widespread platforms as DETECTR [6] and HOLMES [10]. These platforms use short (10–20 nt) ssDNA reporters that are added to the reaction mix. The parameters of the ssDNA reporter for optimal cleavage in a solution were thoroughly examined. The length of ssDNA for maximal cleavage was established by Lv et al. to start at 8 nt, whereas longer molecules showed the same cleavage efficiency [11]. Nucleotide content also impacts cleavage efficiency. It increases in the row: polyT < polyA < polyC. PolyG and G-rich reporters demonstrate either very low cleavage or its absence [11,12].

The release of immobilized ssDNA reporters can be integrated with different biosensing tools to enhance and register generated signals, such as the use of enzymes, nanoparticles, etc. [13,14,15,16]. The proposed biosensors use ssDNA reporters with different lengths (10–200 nt) and sequences [17,18,19,20], or else use more complex reporters such as DNA hairpins and ds/ssDNA composites [21,22,23,24]. A few works have considered the comparison of immobilized DNA of different lengths. For instance, Fu et al. tested four polyT ssDNA immobilized on 13 nm of gold nanoparticles and found a bell-shape dependence with an optimum in the range of 15–25 nt [25]. In contrast, Dai et al. did not find any differences for 10–30 nt ssDNA immobilized on plain gold electrodes [17]. In the case of ds/ssDNA composites immobilized on magnetic particles (MPs), the optimal length of the dsDNA component located between the surface and 15 nt polyT ssDNA was found to be equal to 120–300 bp [26]. To the best of our knowledge, no study has been reported to have found the most efficient trans-activity of Cas12 as it relates to immobilized ssDNA reporters of different lengths and nucleotide content. The existing data considered above indicate that the optimal trans-target parameters of the attached ssDNA may differ from those of ssDNA in solution. This difference is anticipated due to at least two factors: steric hindrance for Cas12a and conformation changes for ssDNA near the surface.

In response to the above considerations, this study presents the comparison of polyA, polyC, and polyT ssDNA of different lengths as trans-target reporters immobilized on MPs. We found that length-dependent effects differed for various kinds of ssDNA. We first demonstrate that the dependence of cleavage efficiency on nucleotide content differs from that obtained for unattached ssDNA and increases in the row: polyA < polyT < polyC. Second, we demonstrate that using polyC and polyT probes with an optimal length provides sensitive detection of DNA targets in two examples of bacterial pathogens: *Dickeya solani* (causes blackleg and soft rot) and *Erwinia amylovora* (causes fire blight). Finally, we demonstrate the efficiency of signal amplification upon the addition of a nanozyme (Au@Pt)–polyA conjugate to a MPs–polyT conjugate after trans-cleavage.

## 2. Materials and Methods

### 2.1. Materials

Two commercial paramagnetic iron microparticle preparations covered with streptavidin (MPs) were used—SpeedBeads magnetic-streptavidin-coated particles (Cytiva, Marlborough, MA, USA) and SiMAG–Streptavidin (Chemicell, Berlin, Germany). Oligonucleotides with modifications (6-carboxyfluorescein (FAM), 5-carboxyrhodamine-X (ROX), biotin, black hole quencher-2 (BHQ2)) were synthesized using Syntol (Moscow, Russia), Evrogen (Moscow, Russia), Lumiprobe (Moscow, Russia) and are presented in Table S1, Supplementary Materials. EnGene LbCas12a, T7 RNA polymerase, DNAseI, NTP, Monarch DNA gel extraction kit, RNA purification kit, and RNAse inhibitor were purchased from NEB (Ipswich, MA, USA). Tersus polymerase and dNTP were obtained from Evrogen. Analytical-grade pure salts and organic compounds were used.

### 2.2. Syntheses of dsDNA Targets for Activation of Cas12a (Cis-Targets)

Two cis-targets for recognizing gRNA–Cas12a were used: ribosomal intergenic spacer (IGS) from *D. solani* and recombinase A (RecA) from *E. amylovora* (sequences are presented in Section S2, Supplementary Materials). The dsDNA fragments (596 bp of IGS, 432 bp of RecA) were amplified for further application using PCR according to protocols described in [27,28]. Details of the syntheses are described in Section S2, Supplementary Materials.

### 2.3. Syntheses of gRNAs

The design, syntheses, and purification of gRNA for recognition of IGS of *D. solani* and gRNA for recognition of RecA of *E. amylovora* are precisely described in [26,28]. See also Section S3, Supplementary Materials, for more details regarding gRNA syntheses.

### 2.4. Conjugation of ssDNA Reporters with MPs

The commercial MPs covered with streptavidin were characterized using transmission electronic microscopy (TEM) and dynamic light scattering (DLS) (Section S4, Supplementary Materials). These MPs (1% *w*/*v*, 2 μL) were conjugated with 30 µL of 100 nM of biotin/FAM-labeled ssDNA reporters (polyT with lengths of 10, 15, 20, 25, 30, 50, 82, and 145 nt, and polyA and polyC with lengths of 10, 30, 40, 50, and 80 nt) and shaken (45–50 rounds/min) for 10 min at 37 °C. Unbound ssDNAs were removed via separation of the MPs using a magnetic holder (Evrogen, Moscow, Russia). For the DNA—MP conjugate pellet was washed using NEB2.1 buffer (NEB, Ipswich, MA, USA) three times and then used for cleavage. The 30 µL of each sample (the initial target DNA solutions (total), or supernatants with unbound DNA (supernatant), or ssDNA–MP conjugates resuspended in NEB2.1 buffer) was mixed with 70 µL of 25 mM Tris–HCl, pH 8.0, with 50 mM of NaCl (F-buffer). After that, the intensity of the FAM fluorescence (I) was measured using black 96-well Fluoro Nunc microplates (Thermo Scientific, Waltham, MA, USA) and an EnSpire multimode plate reader (PerkinElmer, Waltham, MA, USA) with an excitation wavelength of 498 nm and an emission wavelength of 517 nm. The number of flashes was 1000. The loading of MPs was estimated as (I_total_ − I_supernatant_)/I_total_ within each set of measurements of trans-activity.

### 2.5. Synthesis of Au@Pt Nanozyme and Its Conjugate with PolyA-80

The synthesis of Au@Pt nanozyme was carried out as proposed in [29]. Methods of the synthesis of the nanozyme and its conjugate with polyA-80 are described in detail in Section S5, Supplementary Materials.

### 2.6. Trans-Cleavage of ssDNA Reporters Attached to MPs Using Cas12a

Initially, a gRNA–Cas12a premix was prepared following the New England Biolabs (NEB) guidelines with some alterations. The mix contained 66 nM of each gRNA and EnGene LbCas12a, both blended in NEB2.1 buffer. The mix was incubated at 25 °C for 10 min. Subsequently, 3.3 nM of dsDNA cis-target (either IGS or RecA) was added to the mix and incubated for 30 min at 37 °C. Afterward, a ROX–dT15–BHQ2 probe (500 nM, 1 µL) was added as a control for the Cas12a catalytic activity. The activated Cas12a reaction mix (30 µL) was then introduced to the ssDNA–MP conjugate pellet (2 µL, 1% MPs, 100 nM DNA). The reaction was allowed to proceed while being agitated at 37 °C for another 30 min. The reaction was stopped by adding ethylenediaminetetraacetic acid (EDTA) to a final concentration of 25 mM. Following this, the ssDNA–MP conjugate and the cleaved-off ssDNA with fluorescein (ssDNA–FAM) were separated using a magnetic holder. The resulting supernatant was combined with 70 µL of F-buffer. The samples were placed in a black microplate and the readings were taken with an EnSpire multimode plate reader (PerkinElmer, Waltham, MA, USA). The fluorescence of FAM and ROX was assessed for their excitation and emission wavelengths (FAM: λ_ex_ 498 nm, λ_em_ 517 nm; ROX: λ_ex_ 578 nm, λ_em_ 604 nm). Finally, the data obtained were statistically analyzed using OriginProLab 11 software (OriginLab, Northampton, MA, USA).

### 2.7. Detection of dsDNA Targets Using CRISPR–Cas12 Assay in a Homogeneous Format

For the homogeneous assay, NEB2.1 buffer with 66 nM of gRNA and 66 nM of LbCas12a was mixed and incubated at 25 °C for 10 min. Then, ROX–dT15–BHQ2 probe was added to a final concentration of 500 nM in solution with the Cas12a–gRNA complex. The dsDNA target (IGS, RecA) dilutions were manufactured in the concentration range from 10 nM to 4 pM. The cleavage reaction began upon the addition of the 3 µL sample with the dsDNA target at 37 °C. The total volume of the reaction was 30 μL. The ROX fluorescence was measured every 30 s with a Light Cycler 96 (Roche, Rotkreuz, Switzerland). The values that accord to three standard deviations of the null sample signal were considered to be the detection limits.

### 2.8. Detection of dsDNA Targets Using CRISPR–Cas12 Assay in a Heterogeneous Format

For the heterogeneous assay, the procedure completely coincided with that described in Section 2.6. The polyT-82 and polyC-40 ssDNA reporters immobilized on the MPs were used as optimum ssDNA reporters. Instead of 3.3 nM of dsDNA cis-target, a concentration ranging from 4 pM to 10 nM was used for IGS or RecA targets.

For analysis with polyT-82, an additional amplification step was performed. After trans-cleavage and removal of the supernatant, the Au@Pt nanozyme conjugated with polyA-80, corresponding to 1, 3, or 10 nM, was added to the final concentration of polyA-80 in the vial with the MP–polyT-82 conjugate precipitate. The mixture of the MP–polyT-82 conjugate and Au@Pt–polyA-80 was incubated, stirring for 10 min at 37 °C. Next, the resulting MP–polyT-82/Au@Pt–polyA-80 complexes were separated from the unbound Au@Pt–polyA-80 (or streptavidin–peroxidase–polyA-80) using a magnetic holder. The 100 µL of ready-to-use 3,3′,5,5′-Tetramethylbenzidine (TMB)-based substrate solution for peroxidase (Immunotech, Moscow, Russia), containing H_2_O_2_ and an extra 20 µL of 30% H_2_O_2_, was added to the pellet [30]. After 15 min of incubation at room temperature, the reaction was stopped by the addition of 50 µL of 1 M H_2_SO_4_. The signal was recorded using a Zenyth 3100 (Anthos Labtec Instruments, Wals, Austria) microplate spectrophotometer at a wavelength of 450 nm (A_450_). The signal for concentration plotting was obtained as the modulus of the difference between the reaction at zero concentration of cis-target and the reaction at the tested concentration of cis-target.

For concentration dependences, with fluorescence detection of released FAM with colorimetric detection of TMB oxidation with the Au@Pt nanozyme, the detection limits were equaled to values using a sigmoidal fitting method that accorded to three standard deviations of the null sample signal were considered as detection limits.

## 3. Results

### 3.1. Design of Experiments

**Figure 1:**
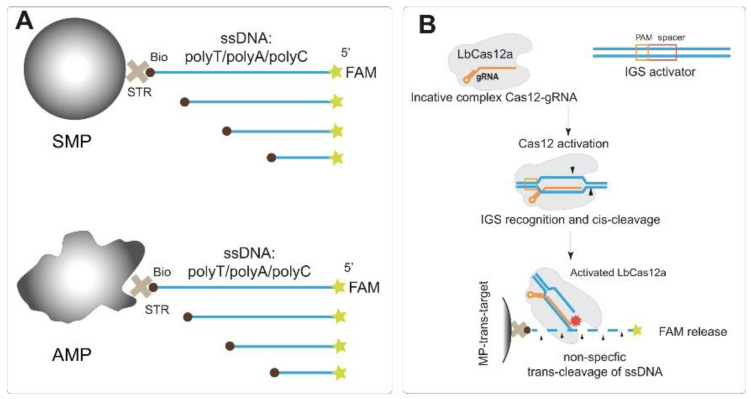
Scheme of experiments. (**A**) biotin/FAM labeled ssDNA reporters conjugated with two MPs: symmetrical shape (SMP) and asymmetric shape (AMP). (**B**) Transcleavage of immobilized ssDNA reporters for IGS target detection (dsDNA target of ribosomal intergenic spacer from *D. solani*). STR—streptavidin, Bio—biotin, FAM—fluorescein, MP—magnetic particle.

We chose three types of homopolymer ssDNA to test their trans-cleavage—polyT, polyA, and polyC. The use of homopolymers provides a complete absence of double-stranded structure formation within the ssDNA molecule. Each type was presented by sets of oligonucleotides with different lengths. Every ssDNA preparation was 5′-labeled by FAM and 3′-labeled by biotin. As well as in previous studies [6,31], the effect of the label position on trans-cleavage was not observed; we did not vary the FAM/biotin position. PolyT ssDNA with 10, 15, 20, 25, 30, 50, 82, and 145 nt were tested. PolyA and polyC were presented with a range of lengths corresponding to 10, 20, 30, 40, 50, and 80 nt. PolyG is very difficult to synthesize longer than 5–6 nt, and we decided to omit the homopolymer in our research. The approximate physical lengths of the studied ssDNA molecules correspond to the range from 6.7 to 96.7 nm (more detailed data about the number of nucleotides and the lengths of oligonucleotides are presented in Table S2, Supplementary Materials). Conjugation of the ssDNA reporters with MPs was performed by the interaction of biotin with streptavidin-covered surface of the MPs (Figure 1A).

As carriers for ssDNA reporters, we used MPs due to the widely applied possibility of their simple and rapid separation in magnetic fields. The integration of the MP-based technique with Cas12a-based biosensors presents a clear benefit. This combination allows for convenient manipulation of both noncleaved DNA and released labeled DNA. In this study, two types of MPs with covalent-bound streptavidin were conjugated with ssDNA: ones with a symmetrical spherical shape (named SMP) and possessing a mean hydrodynamic diameter equal to 977.6 ± 106.7 nm, and asymmetric MPs (AMP) possessing a mean hydrodynamic diameter equal to 1498.0 ± 154.9 nm (see TEM images in Figure S1, distributions of hydrodynamic diameters at Figure S2, Supplementary Materials). The use of two types of MPs allows us to exclude specific features of some preparations from an interpretation of the trans-cleavage dependences. Streptavidin–biotin is a well-studied receptor–ligand pair that provides simple and high-affinity binding [32]. In addition, chosen MP-covered streptavidin is suitable for providing rapid and quite stable capture of biotin-labeled oligonucleotides with high capacity, as previously shown [26,33]. The immobilization of ssDNA was carried out under conditions providing optimal loading (i.e., maximal binding) of biotinylated DNA on the MP-covered streptavidin [26]. Fluorescent signals of the ssDNA reporters were measured before immobilization, and in unbound ssDNA solutions, to calculate the degree of loading (%).

The Cas12a–gRNA complex was activated by the dsDNA target (IGS fragment of *D. solani* or RecA fragment of *E. amylovora*), and the activated Cas12a–gRNA–dsDNA complex was added to the MPs–ssDNA conjugates (Figure 1B). After trans-cleavage, the released FAM was separated, and the fraction of released FAM was calculated and considered as a measure of cleavage efficiency. The detection of trans-cleavage of the ssDNA immobilized on the MP surface was performed in two ways: (1) based on the fluorescence of the released FAM and (2) using the chromogenic reaction of TMB oxidation in the presence of Au@Pt nanozyme conjugated with polyA, which was bound by MPs–polyT due to polyT/polyA interactions.

### 3.2. Trans-Cleavage of Different Types of ssDNA Immobilized on MPs

To compare ssDNA of different contents and lengths, an IGS dsDNA cis-target was used for the activation of Cas12a. Primarily, the effect of the surface density of ssDNA on trans-cleavage was tested on the polyT-15 trans-target when the concentration of MPs was constant (0.0625%) and the concentration of polyT-15 was varied from 25 to 200 nM. The loading and trans-cleavage efficiency were not dependent on the trans-targets: MP ratio (Figure S5, Supplementary Materials). Therefore, the chosen concentrations (100 nM of ssDNA and 0.0625% of MPs) were in the optimal range.

**Figure 2:**
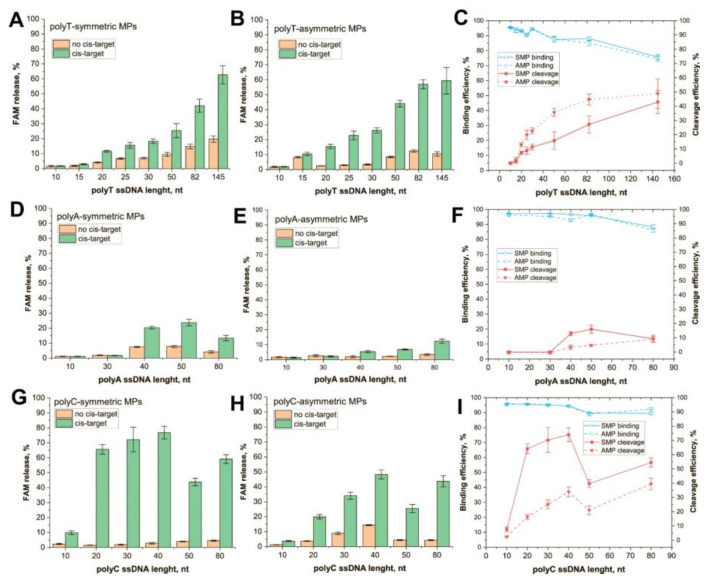
Trans-cleavage of ssDNA immobilized on MPs. FAM release for (**A**) SMP-polyT, (**B**) AMP-polyT, (**D**) SMP-polyA, (**E**) AMP-polyA, (**G**) SMP-polyC, and (**H**) AMP-polyC. Efficiency of cleavage and binding with MP for (**C**) polyT, (**F**) polyA, and (**I**) polyC.

Conjugated polyT–ssDNA demonstrated a gradual increase in cleavage efficiency for both SMP and AMP as carriers (Figure 2A,B). Internal control shows high activity (>50% of the cleaved reporter) of Cas12a in all experiments with the cis-activator, and activity not exceeding 5% in experiments without the activator (Figure S6A,B, Supplementary Materials). Note that the FAM release, in the absence of a cis-target, increased for longer ssDNA. This could be caused by two factors: (1) dissociation of ssDNA that were bound to the MP’s surface by many weak nonspecific interactions between DNA backbone/residues and the MP or streptavidin, (2) non-enzyme degradation of ssDNA. For short ssDNA, this effect was also observed and was associated with nonspecific sorption on MP surface, for example, polyT-15 and AMP (see Figure 2B). Indeed, longer ssDNA has a higher probability of spontaneous hydrolysis. After subtracting nonspecific responses, dependence of cleavage efficiency from the length of the polyT ssDNA demonstrated a hyperbola shape (Figure 2C, red lines). Immobilization of AMPs caused more efficient cleavage as compared with SMPs, and the maximal cleavage efficiency (40–50%) was reached for AMP conjugates with 50 nt polyT. In the case of AMPs, a slow increase in the efficiency was observed since 50 nt polyT molecules. The binding capacity for both MPs is slightly decreased for 50, 82, and 145 nt polyT ssDNA (Figure 2C, blue lines).

PolyA conjugates revealed a low FAM release starting from 40 nt ssDNA (Figure 2D,E). The efficiency of cleavage was bell-shaped for SMPs with a maximum (16%) for 50 nt ssDNA and linear for AMPs (Figure 2F, red lines). Both kinds of conjugates showed low activity. In the case of AMP, maximal efficiency (9%) was reached by 80 nt ssDNA. The ROX–dT–BHQ2 probe as an internal control was found to be inappropriate because its 15 nt polyT region forms complementary dsDNA with the polyA ssDNA. Therefore, to prove the activity of Cas12a in the reaction, we performed the control reaction in parallel. Cas12a cleavage of 30 nt unbound FAM/biotin-labeled polyA ssDNA was performed under the same conditions as in the experiments with the MP–polyA-30 ssDNA. After the reaction SMPs were added to bound uncleaved ssDNA. The experiment showed a high amount of released FAM comparing the Cas12a treatment without cisactivator (Figure S6, Supplementary Materials). It proves that Cas12s can effectively cleave 30 nt polyA in the solution but not its immobilized form. Interestingly, the cleavage of unbound polyA was 25% less effective than the cleavage of polyT with the same length. That observation is not consistent with the data of predecessors that compared cleavage in a solution of ssDNA with different compositions [11,12].

PolyC ssDNA conjugates showed high (50–70%) release of FAM for SMPs as carriers (Figure 2G) and moderate (20–50%) release for AMPs (Figure 2H). Low activity was detected even for 10 nt polyC. The growth of cleavage efficiency continued up to 40 nt polyC for both MPs (Figure 2I, red lines). Internal control proved the high activity of Cas12a in each sample with the cis-activator (Figure S6C,D, Supplementary Materials).

The implemented comparison of polyT, polyA, and polyC, and comparison with earlier studies, allow us to state the following dependencies:

1. PolyC ssDNA is the most effective trans-target that compares polyA or polyT of the same length (see Figure 2C,F,I). This feature is more expressed for SMP conjugates. It agrees with data from previous research that establish polyC as being the most effective trans-target in the solution. In our research, the effectivity of cleavage increases in the row polyA < polyT < polyC, wherein, the cleavage of each immobilized ssDNA was less effective than the cleavage of the corresponding free ssDNA (Figures S6 and S7). This can be caused by steric hindrance and/or restricted diffusion of Cas12s near the MP surface. In experiments in which polyT was immobilized on the gold layer, the cleavage efficiency was 20–25% of its maximum value [17]. Meanwhile, conjugate polyT, with small (13 nm) GNPs, demonstrated the same cleavage efficiency as unbound polyT [25]. As such, it can be expected that the size of carrier particles influences the extent of the decrease in cleavage efficiency.
2. No linear dependence of efficiency was observed from trans = target length. The dependencies had hyperbolic saturation or a bell shape. The minimum length for the cleavage was determined using the type of homopolymers, following the row polyC < polyT < polyA. Thus, the most effective homopolymer for the cleavage of the shorter oligonucleotide can be used. At the same time, the length for maximally effective cleavage (20–50 nt) is significantly higher for MP-bound targets than for soluble trans-targets (8 nt) [11]. This could be a result of steric and/or charge effects of the MP surface. An AMP with a more heterogenous surface showed less efficient trans-cleavage.
3. There was no unambiguous increase in cleavage efficiency with increasing ssDNA length. Limitations for long ssDNAs can be explained due to the loss of linear structure. Curving trends appear even for very short ssDNAs, starting from 0.7–6 nm in length [34,35]. Pyrimidine polymers are more flexible; for some lengths, they tend to form compact globules [36]. Unlike them, polyA oligonucleotides tend to form rod conformations that resemble dsDNA due to adenine stacking [37,38]. This property of polyA ssDNAs would potentially require an increase in their accessibility to Cas12a, but we did not observe it. In the case of SMPs, some interactions of the nucleotides with the MP surface could lead to lower cleavage efficiency for long ssDNAs. Moreover, the binging of long ssDNA to MPs is worse than short ones.

Integrating the given above analysis, we recommend using 20–40 nt polyC reporters for the immobilization on magnetic particles. This choice provides the most effective cleavage. Note that, actually, Cas12a-based biosensors with surface-attached ssDNA reporters used polyA and polyT, but not polyC without prior comparative studies—see [19,20,21,22,23,24,39,40]. Collected key characteristics of these biosensors are presented in Table S3, Supplementary Materials.

### 3.3. Detection of dsDNA Targets Using CRISPR–Cas12 Assay in Homogeneous and Heterogeneous Formats

To estimate the efficiencies of ssDNA reporters in a heterogeneous assay with Cas12a, we carried out the detection of dsDNA targets that match *D. solani* and *E. amylovora*. We used two ssDNA reporters attached to MPs: (1) polyC-40 (attached to SMP, see Figure 2G) as an optimal ssDNA reporter, and (2) polyT-82 (attached to AMP, see Figure 2B) as a commonly used reporter providing hybridization with polyA tails with accession of the nanozyme that generate detected signals.

**Figure 3:**
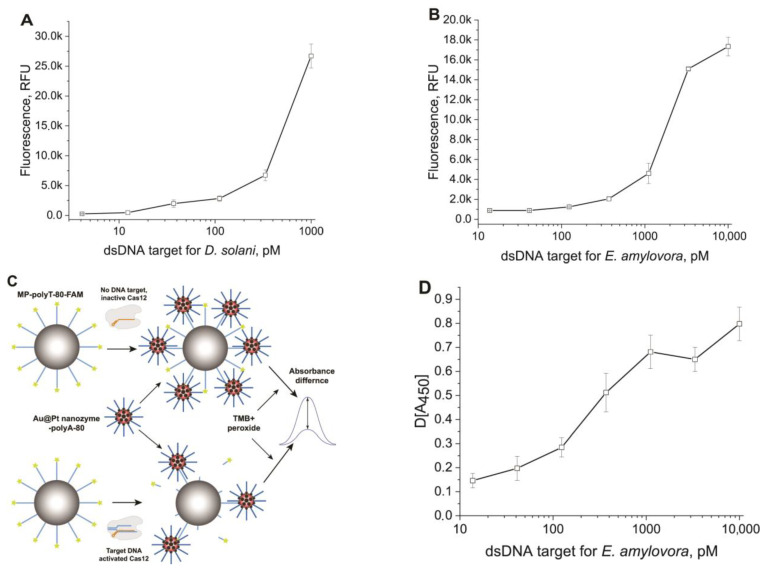
Detection of dsDNA targets using CRISPR–Cas12a assay in heterogeneous format. (**A**) Concentration dependence for dsDNA target *D. solani*, obtained with MP–polyC-40 and fluorescence detection of released FAM (sigmoid function with parameters: bottom = 845.0, top = 29,112, IC50 = 566.7, HillSlope = 1.92; R^2^ = 0.99711). (**B**) Concentration dependence for dsDNA target of *E. amylovora* obtained with MP–polyT-82 and fluorescence detection of released FAM (sigmoid function with parameters: bottom = 846.5, top = 0.4, IC50 = 1720.2, HillSlope = 1.61; R^2^ = 0.99711). (**C**) Scheme of amplification based on Au@Pt–polyA binding with MPs–polyT conjugate after transcleavage. (**D**) Concentration dependence for dsDNA target of *E. amylovora* obtained with MP–polyT-82 and colorimetric detection of TMB oxidation by the Au@Pt nanozyme (sigmoid function with parameters: bottom = 0.14, top = 0.72, IC50 = 263.5, HillSlope = 1.35; R^2^ = 0.95491). The signals are the modulus of the difference between the response at zero concentration of the cistarget and the response at the tested concentration of the cistarget.

For the detection of the dsDNA target *D. solani* (IGS), we developed its serial dilutions and tested them in a heterogeneous format (see Section 2.8), and for comparison in a homogeneous format (see Section 2.7). The detection of the fluorescence of the released FAM after trans-cleavage of MP–polyC-40 provided the concentration dependence for the dsDNA target of *D. solani* (Figure 3A). The limit of detection was 20 pM, which corresponded to the results that were obtained in a homogeneous Cas12a-based assay with ROX–15dT–BHQ2 probe (Figure S8A, Supplementary Materials).

To detect dsDNA target *E. amylovore* (RecA), we developed its serial dilutions and tested them in heterogeneous and homogeneous formats. The obtained concentration dependence is presented in Figure 3B. The limit of detection for the dsDNA target *E. amylovora* was 50 pM. Thus, the heterogeneous assay was about three times less sensitive than the homogeneous Cas12a-based assay with the ROX–15dT–BHQ2 probe (Figure S8B, Supplementary Materials).

Thus, both heterogeneous assays with different ssDNA reporters showed results close to the homogeneous assays. However, the system with the 40 nt polyC was expected to be somewhat more sensitive (see data about cleavage efficiency in Section 3.2). To improve the sensitivity of CRISPR–Cas-based biosensors, various nucleic acid-based signal amplification techniques were incorporated [41]. The use of CRISPR–Cas biosensors without prior nucleic acid amplification simplifies the diagnostic process and reduces the time and resources required for accurate analyte detection [42,43]. At the same time, the advantage of MP-based heterogeneous systems is the possibility of introducing alternate labels with more sensitive detection. To demonstrate this concept, we used an Au@Pt nanozyme with peroxidase-like properties. The scheme of the amplified assay using these conjugates and polyA-80 is shown in Figure 3C. The given reactants are added to the pellet containing MP–polyT-82 after trans-cleavage. The following polyA/polyT interactions led to particles binding and detection of even a slight cleavage of polyT on the MP surface. We assumed that the products of trans-cleavage do not interfere with the binding of polyA-80 on the Au@Pt nanoparticle surface and do not compete with polyT-82, causing a false-positive signal. First, this is because most of the cleaved ssDNA is separated from the MP conjugate and removed before adding polyA-80–Au@Pt. Secondly, cleavage in the solution produces short (2–4 nt) oligonucleotides [31]. Moreover, we assumed that any released ssDNA fragment from polyT-82 was shredded in the solution until 2–4 nt fragments (based on data in Figure S6, Supplementary Materials, and a previous study [26]). Therefore, only a small number of short DNA is present while Au@Pt–polyA-80 is added to the MP conjugate. The predicted dissociation constants for different lengths of the polyT/A duplex were high for 2–4 polyT/A duplexes and should be displaced from polyT-82 by most possible frames of interaction between polyA-80 and polyT-82 (Figure S10, Supplementary Materials).

The Au@Pt nanozyme with a hydrodynamic diameter of 106.7 nm, synthesized on the basis of 26.1 nm (see Figure S4, Supplementary Materials) Au nanoparticles, showed strong peroxidase-like properties in the reaction with TMB, both before and after its coating with streptavidin (testing of its catalytic properties is presented in Section S8, Supplementary Materials). Nanozyme–streptavidin conjugate was used for coupling with biotinylated polyA-80 (see methods, TEM images, and distributions of hydrodynamic diameters in Section S5, Supplementary Materials). Concentration dependencies for the interactions with MP–polyT-82 and polyA-80 of two catalytically active conjugates were fundamentally different (see Figure S9B, Supplementary Materials), which influenced their optimal concentrations. The chosen value was 3.3 nM for Au@Pt–streptavidin–polyA-80 (molarity accorded to the content of polyA-80 in the conjugate). We analyzed the MP–polyT-82 conjugate after trans-cleavage in reaction with different concentrations of the dsDNA cis-target of *E. amylovora*. The use of the Au@Pt–streptavidin–polyA-80 provided specific signals with a pronounced concentration dependence in the range from 14 to 10,000 pM of the cis-target (Figure 3D).

Thus, the presented nanozyme-based development demonstrates success in the amplification approach when combined with Cas12a-based biosensors that use ssDNA probes attached to MPs. This direction has great potential for the further development of highly sensitive biosensors.

## 4. Conclusions

Finding the most efficient ssDNA probes enables the development of sensitive Cas12a-based heterogeneous biosensors, wherein the cleavable ssDNA trans-target probe is connected to the carrier surface. The objective of our study was to analyze polyA, polyC, and polyT ssDNA probes of varying lengths (ranging from 10 to 145 nt), which were immobilized on magnetic particles. First, significant differences between the probes were demonstrated. Among ssDNAs of equal length, polyC was the most effective for Cas12 cleavage. The impact of the nucleotide content on the cleavage efficiency differs from the case of soluble ssDNA probes and demonstrates escalation in the row polyA < polyT < polyC. The effectiveness of polyC and polyT probes of optimal length was proven in a heterogeneous Cas12a-based assay for DNA targets of phytopathogens *Dickeya solani* and *Erwinia amylovora*. Additionally, we demonstrated an amplified Cas12a-based heterogeneous assay with an increased signal through the combination of polyA-covered Au@Pt nanozyme and trans-cleaved MPs–polyT.

These insights pave the promising pathways for Cas12a-based heterogeneous assays of viruses, bacteria, and other DNA/RNA-containing analytes. These findings could significantly enhance the detection methods and contribute to the evolving field of biosensors.

## Data Availability

The data presented in this study are available on request from the corresponding author.

## Supplementary Materials

The following supporting information can be downloaded at: https://www.mdpi.com/article/10.3390/bios13070700/s1, Figure S1: TEM images of AMPs and SMPs; Figure S2: Characterization of streptavidin–MPs and their conjugates with biotinylated ssDNAs using DLS; Figure S3: TEM images of Au NPs and Au@Pt NPs; Figure S4: Characterization of Au NPs, Au@Pt NPs, Au@Pt–streptavidin conjugate, and Au@Pt–streptavidin–polyA-80 conjugate using DLS; Figure S5: Binding and cleavage of poly-dT-15 upon different concentrations of the targets and constant concentration of MPs; Figure S6: Control measurements of ROX–15dT–BHQ2 probe cleavage; Figure S7: Control measurements of polyA and polyT probe cleavage with following conjugation; Figure S8: Fluorescence plots of a cleaved ROX–dT15–BHQ2 probe in results of detection of dsDNA targets using CRISPR–Cas12 assay in homogeneous format; Figure S9: Estimation of different dilutions of Au@Pt–streptavidin–polyA-80 conjugate in the reaction with TMB-based substrate solution for peroxidase; Figure S10: Dependences of predicted Kd on polyT–A length based on different NN-models; Table S1: Sequences of the primers and oligonucleotide probes for DNA constructs used in this research; Table S2: Length of ssDNA reporter; Table S3: ssDNA oligonucleotides used in previous research. References [44,45] are cited in the Supplementary Materials.
