# Supplementary material for: Comparison of Single-Stranded DNA Probes Conjugated with Magnetic Particles for Trans-Cleavage in Cas12a-Based Biosensors
Source: Biosensors (Basel). 2023 Jul 1;13(7):700. doi: 10.3390/bios13070700 (PMC10376970; doi:10.3390/bios13070700)
Supplement: Supplementary file 1 [file biosensors-13-00700-s001.zip › biosensors-2445885-supplementary.pdf]

Supplementary Materials

Comparison of single-stranded DNA probes conjugated with magnetic particles for trans-cleavage in Cas12a-based biosensors

Aleksandr V. Ivanov[1], Irina V. Safenkova[1] *, Anatoly V. Zherdev[1], Yi Wan[2] and Boris B. Dzantiev [1]

CONTENT

Section S1. Characteristic of using oligonucleotides 2

Section S2. Synthesis of dsDNA targets for activation of Cas12a (cistargets) 4

Section S3. Synthesis of guide RNAs 5

Section S4. Characterization of streptavidin–MPs. 6

S4.1. Characterization of streptavidin–MPs using transmission electron microscopy (TEM) 6

Section S5. Synthesis and characterization of Au nanoparticles, Au@Pt nanozyme, conjugate with polyA-80 8

S5.1. Synthesis of Gold Nanoparticles 8

S5.2. Synthesis of Au@Pt Nanoparticles 8

S5.3. Synthesis of Au@Pt Nanoparticles Conjugates with polyA-80 8

Section S6. Transcleavage of immobilized ssDNA 10

Section S7. Attached ssDNA oligonucleotides that were used in previous research 12

Section S8. Detection of dsDNA targets using CRISPR–Cas12 assay in homogeneous format 13

Section S9. Characteristic of Au@Pt–streptavidin–polyA-80 conjugate 14

Section S1. Characteristic of using oligonucleotides

Table S1. Sequences of the primers and oligonucleotide probes for DNA constructs used in this research.

Name	Sequence 5′-3′	Purpose
M13 F	GTTGTAAAACGACGGCCAGTG	Synthesis of IGS-target with 596 bp length
M13 R	AGCGGATAACAATTTCACAC	
gRNA1 IGS F	TTTTTTTTAATACGACTCACTATAGGTAATTTCTACTAAGTGTAGA-TAAACGACGGCATGCGAGCGTG	Transcription of gRNA for IGS-target
gRNA1 IGS R	CACGCTCGCATGCCGTCGTTTATCTACACTTAGTAGAAATTAC-CTATAGTGAGTCGTATTAAAAAAAA	
RecA F	CCGCACAGCGTAAAGGTAAG	Synthesis of RecA-target with 432 bp length
RecA R	GAATATCCAGACGGACAGAG	
gRNA1 RecA F	TTTTTTAATACGACTCACTATAGGTAATTTCTACTAAGTGTAGAT-TCAACCAGATCCGTATGAAAAT	Transcription of gRNA for RecA -target
gRNA1 RecA F	ATTTTCATACGGATCTGGTTGAATCTACACTTAGTAGAAATTAC-CTATAGTGAGTCGTATTAAAAAA	
ROX-dT15-BHQ2	ROX-TTTTTTTTTTTTTTT-BHQ2	Internal control of trans-cleavage
polyT10	FAM-TTTTTTTTTT-Bio	Trans-cleavage
polyT15	FAM-TTTTTTTTTTTTTTT-Bio	
polyT20	FAM-TTTTTTTTTTTTTTTTTTTT-Bio	
polyT25	FAM-TTTTTTTTTTTTTTTTTTTTTTTTT-Bio	
polyT30	FAM-TTTTTTTTTTTTTTTTTTTTTTTTTTTTTT-Bio	
polyT50	FAM-TTTTTTTTTTTTTTTTTTTTTTTTTTTTTTTTTTTTTTTTTTTTTTTTTT-Bio	
polyT82	FAM-TTTTTTTTTTTTTTTTTTTTTTTTTTTTTTTTTTTTTTTTTTTTTTTTTTTTTTTTTTTTTTTTTTTTTTTTTTTTTTTTTT-Bio	
polyT145	FAM-TTTTTTTTTTTTTTTTTTTTTTTTTTTTTTTTTTTTTTTTTTTTTTTTTTTTTTTTTTTTTTTTTTTTTTTTTTTTTTTTTTTTTTTTTTTTTTTTTTTTTTTTTTTTTTTTTTTTTTTTTTTTTTTTTTTTTTTTTTTTTTTTT -Bio	
polyA10	FAM-AAAAAAAAAA-Bio	
polyA30	FAM- AAAAAAAAAAAAAAAAAAAAAAAAAAAAAA -Bio	
polyA50	FAM-AAAAAAAAAAAAAAAAAAAAAAAAAAAAAAAAAAAAAAAAAAAAAAAAAA -Bio	
polyA80	FAM-AAAAAAAAAAAAAAAAAAAAAAAAAAAAAAAAAAAAAAAAAAAAAAAAAAAAAAAAAAAAAAAAAAAAAAAAAAAAAAAA -Bio	
polyC10	FAM-CCCCCCCCCC-Bio	
polyC20	FAM-CCCCCCCCCCCCCCCCCCCC -Bio	
polyC30	FAM-CCCCCCCCCCCCCCCCCCCCCCCCCCCCCCCCCCCCCCCC -Bio	
polyC50	FAM-CCCCCCCCCCCCCCCCCCCCCCCCCCCCCCCCCCCCCCCCCCCCCCCCCCCCCCCCCCCC -Bio	

polyC80	FAM-<br>CCCCCCCCCCCCCCCCCCCCCCCCCCCCCCCCCCCCCCCCCCCCC<br>CCCCCCCCCCCCCCCCCCCCCCCCCCCCCCCCCCCCCCCCCCCCC<br>CCCCCCCCCC -Bio

* Bio—biotin, FAM—fluorescein, ROX—5-carboxyrhodamine-X, BHQ1—black hole quencher 2.

Table S2. Length of ssDNA reporter.

polyN nucleotides	Length, nm
10	6.67
15	10.00
20	13.34
25	16.66
30	20.01
50	33.35
80	53.36
82	54.70
145	96.72

Section S2. Synthesis of dsDNA targets for activation of Cas12a (cis-targets)

Sequences of 596 IGS cis-targets that correspond to double-strand fragments. IGS region of *D. solani* is colored light grey. Double underline denotes PAM motive (5′-TTTA-3′). Thick underline denotes gRNA recognition site (along complementary strand in this cistarget DNA). To simplify, coding strands, only (5′->3′) are shown.

5′-ACGTTGTAAAACGACGGCCAGTGAATTGTAATACGACTCAC-
TATAGGGCGAATT-
GGGCCCGACGTCGCATGCTCCCGGCCGCCATGGCGGCCGCGGGAATTCGATGAA
AGTGTCTCGGGATGCGGGTATATTGAGAGACTCGACCGGCACACAAACTCAC-
GCTCG-
CATGCCGTCGTTTCAAATTATTCAGCTTGTTCCGGATTGTTAAAGAGCAGATAACA
TAAACCTGACTATCTCTAATCAGTTTTAGGTTAGCGTTGAC-
CGTGCCTTTCACCCAC-
CGTCAGTCATATTGGCGTCCCCTAGGGGATTCGAACCCCTGTTACCGCCGTGAAA
GGGCGGTGTCCTGGGCCTCTAGACGAAGGGGACATCACTTGTCAGCTTCGCAA-
GACGCTTTT-
GACTCTTTCTTATCATCAGACAATCTGTGTGGACACCACGCAGGCACTTCAAATC
ACTAGTGAATTCGCGGCCGCCTGCAGGTCGACCATATGGGAGAGCTCCCAAC-
GCGTT-
GGATGCATAGCTTGAGTATTCTATAGTGTCACCTAAATAGCTTGGCGTAATCATGG
TCATAGCTGTTTCCTGTGTGAAATTGTTATCCGCT-3′

Sequence of 432 bp recA cis-target that corresponds to double-strand fragments. Double underline denotes PAM motive (5′-TTTA-3′). Thick underline denotes gRNA recognition site (along complementary strand in this cis-target DNA).

5′-CCGCACAGCGTAAAGGTAAGACCTGTGCGTTTATCGATGCCGAGCAC-
GCGCTG-
GACCCGGTTTATGCCAAAAAACTGGGCGTGGACATCGATAATTTACTGTGCTCTC
AGCCAGATACCGGCGAGCAAGCGCTGGA-
GATCTGTGATGCGCTGGCGCGTTCTGGCGCTGTT-
GATGTGATCATTGTTGACTCCGTTGCGGCGTTGACGCCAAAAGCAGAAATCGAG
GGGGAAATCGGCGACTCTCACATGGGCCTTGCGGCGCG-
TATGATGAGCCAGGCAATGCGTAA-
GCTGGCCGGTAACCTGAAAAACTCCGGGACGCTGCTGATCTTTATCAACCAGATC
CGTATGAAAATTGGTGTGATGTTCGGTAACCCGGAAACCACCACCGGCGG-
TAATGCGCTGAAGTTTACGCCTCTGTCCGTCTGGATATTC-3′

The reaction mix (300 μL) contained 200 nM dNTPs, along with 200 nM M13 forward and M13 reverse primers (**Table S1**), 450 ng of pGEM-IGS or pL-*E. amylovora* -recA plasmid, 30 units of Tersus polymerase, and Tersus buffer (Evrogen, Moscow, Russia). PCR was performed within 40 cycles using the BioRad T100 Thermal Cycler (BioRad, Hercules, CA, USA). Each cycle comprises 30 s denaturation at 95 °C, 30 s primer annealing at 55 °C, and then 60 s elongation at 72 °C. The target product was purified via gel electrophoresis in 1.5% agarose in TAE and then extracted using a Monarch DNA gel extraction kit. The IGS contained fragments of full-length IGS (342 bp), and flanked regions came from the plasmid. As a result, the total length of the IGS construct was 596 bp. The PCR product for *E. amylovora* recA comprises 432 bp fragments of only the recA gene.

The concentration of the DNA was estimated using NanoDrop ND-2000 (Thermo Scientific, Waltham, MA USA).

Section S3. Synthesis of guide RNAs

IGS gRNA:
5′-GGUAAUUUCUACUAAGUGUAGAUAAACGACGGCAUGCGAGCGUG – 3′
RecA gRNA:
5′-GGUAAUUUCUACUAAGUGUAGAUUCAACCAGAUCCGUAUGAAAAU – 3′

Site of recognition is denoted with a thick underline.

Complementary oligonucleotides (gRNA DNA F and gRNA DNA R, Table S1), containing T7 promoter genes followed by a 22 bp gRNA gene and a 20-22 bp target DNA fragment, were denatured at 95 °C and then annealed upon, gradually decreasing the temperature to obtain a dsDNA template for in vitro transcription. The reaction mix (100 µL) contained 40 mM Tris–HCl, pH 8.0, 20 mM $MgCl_2$, 2 mM spermidine, 10 mM DTT, 1.25 mM of each NTP, 0.5 u/µL of RNAse inhibitor, 2.5 u/µL of T7 RNA polymerase, and 2 µM of dsDNA template. The reaction was performed at 37 °C for 4 h. To remove residuals of the dsDNA template, 10 µL of DNAseI buffer and 3 U of DNAseI were added and incubated for 30 min at 37 °C. The obtained RNAs were purified using an RNA cleanup kit (NEB) according to the manufacturer's protocol. Then, DNAse treatment and RNA purification were repeated. The concentrations of the gRNAs were estimated using NanoDrop ND-2000 (Thermo Scientific, Waltham, MA, USA).

Section S4. Characterization of streptavidin–MPs.

S4.1. Characterization of streptavidin–MPs using transmission electron microscopy (TEM)

The streptavidin–MPs were characterized using a JEM CX-100 transmission electron microscope (Jeol, Tokyo, Japan) at an accelerating voltage of 80 kV. The glow-discharged 300-mesh copper grid (Ted Pella, Redding, CA, USA) covered with formvar layer was used as a support for TEM. The 2 μL of each of the streptavidin–MP suspensions was diluted in 30 μL of deionized (Simplicity UV, Millipore, Burlington, MA, USA) mQ water. Then, 10 μL was applied on the grid and incubated for 20 min. Excess MPs were removed through mQ rinsing. Typical TEM images for AMPs and SMP are presented in Figure S1.

S4.2. Characterization of streptavidin–MPs and DNA–streptavidin–MP conjugates using dynamic light scattering (DLS)

Hydrodynamic diameters (D_h) of streptavidin–MP particles and their conjugates with ssDNA were measured using a Zetasizer Nano ZSP (Malvern Panalytical, Malvern, UK), which features a 4 mW He–Ne laser (633 nm). An amount of 2 μL of the suspensions was diluted in 200 μL of NEB2.1 buffer (NEB) and inserted into a disposable solvent-resistant microcuvette (ZEN0040; Malvern Panalytical, Malvern, UK). Experimental measurements based on noninvasive backscatter technology were taken with a fixed 173° scattering angle at 25 °C. The experiments were initiated after the sample reached thermal equilibrium. Each sample was measured at least four times, and each measurement consisted of 50 acquisitions. The data were collected and analyzed using Zetasizer Software ver. 8.00. Results are presented in Figure S2.

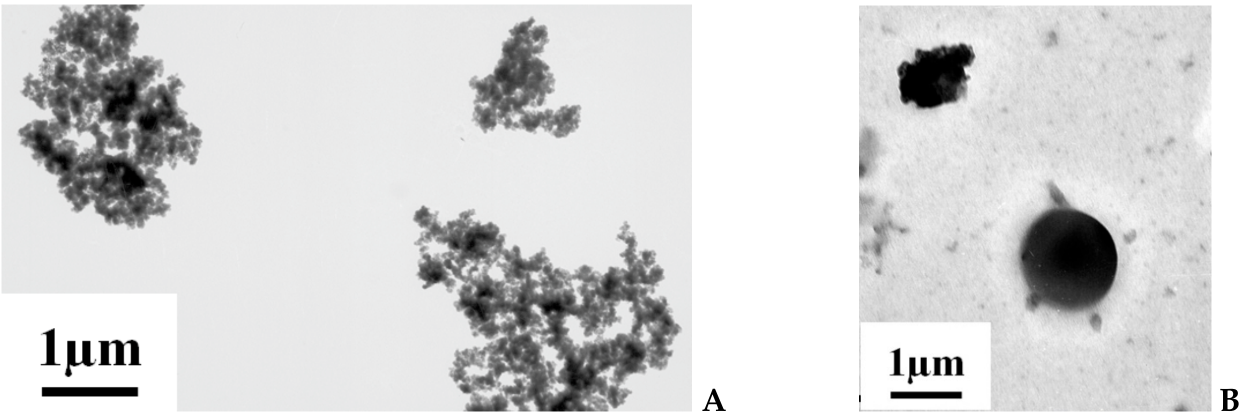

Figure S1. TEM images of AMPs **(A)** and SMPs **(B).**

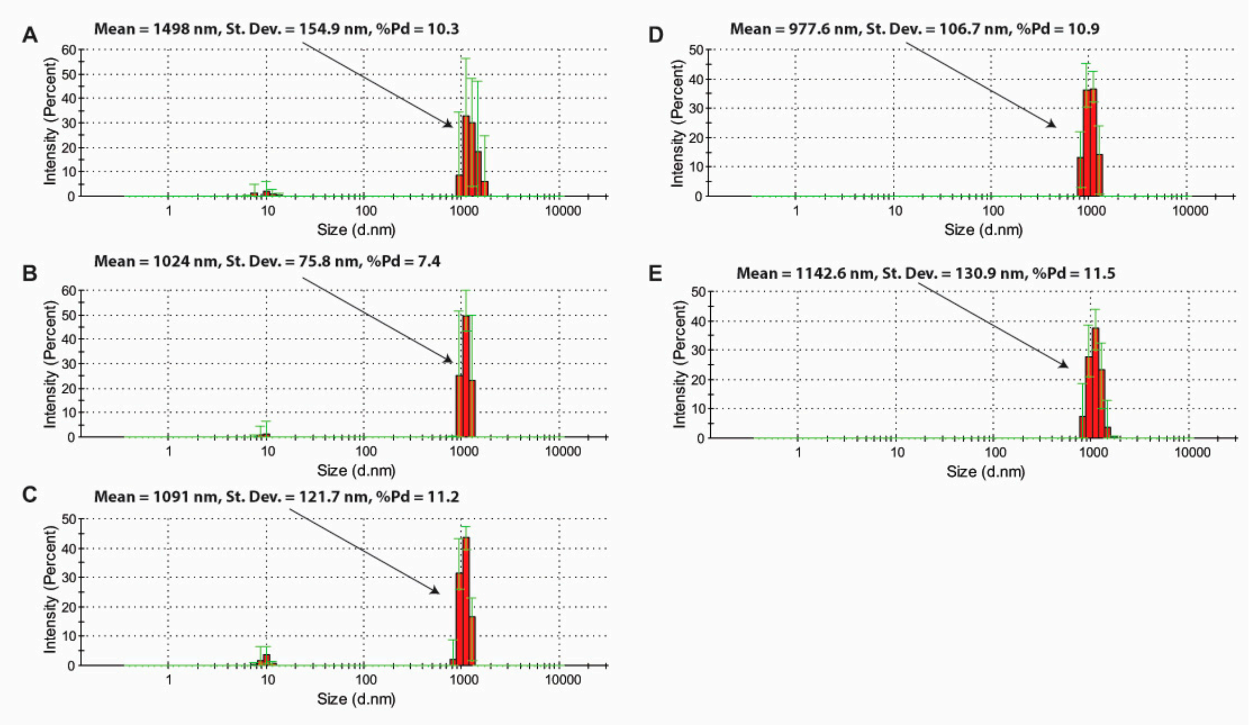

Figure S2. Characterization of streptavidin–MPs and their conjugates with biotinylated ssDNAs using DLS. Distributions of hydrodynamic diameters of AMPs **(A)**, conjugate of AMPs with polyT-80 **(B)**, conjugate of AMPs with polyA-80 **(C)**, SMPs **(D)**, and conjugate of SMPs with polyC-40 **(E)**. Mean—mean hydrodynamic diameters, St. Dev.—standard deviation, %Pd—polydispersity.

Section S5. Synthesis and characterization of Au nanoparticles, Au@Pt nanozyme, and conjugate with polyA-80

S5.1. Synthesis of Gold Nanoparticles

Gold nanoparticles (Au NPs) were synthesized using the Frens method [44], with slight changes. One milliliter of 1% $HAuCl_4$ (Sigma-Aldrich, St. Louis, MO, USA) was added to 95 mL of deionized water. The mixture was continuously stirred and heated to the boiling point; then, 4 mL of 1% sodium citrate was added. The Au NP solution was continuously boiled for another 30 min, then cooled and stored at 4 °C for future use.

S5.2. Synthesis of Au@Pt Nanoparticles

Synthesis of Au@Pt NPs was performed following the protocol described by Panferov et al. [29]. Briefly, 20 mL of 1 nM Au NP solution (see Section S5.1) was mixed with 4 mL of 10 mM Na_2PtCl_6 solution (Sigma-Aldrich, St. Louis, MO, USA) and 5.3 mL of H_2O for 1 min at 80 ± 2 °C. Then, 4 mL of 50 mM sodium ascorbic salt was added at the rate of 400 µL/min using a peristaltic pump. Then, the mixture was stirred for 30 min at 80 ± 2 °C and further stored at 4 °C.

S5.3. Synthesis of Au@Pt Nanoparticles Conjugates with polyA-80

Au@Pt NPs were conjugated with streptavidin (Imtek, Moscow, Russia) through physical adsorption, following the method described by Panferov et al. [29]. The solution of Au@Pt NPs was adjusted to pH 9.5. Then, 12 µg of streptavidin was added to each 1 mL of Au@Pt solution (1 nM). The synthesis was carried out at RT for 1 h, with continuous mixing. Afterwards, BSA (Sigma-Aldrich, St. Louis, MO, USA) was added to a final concentration equal to 0.25%. The mixture was centrifuged at 10,000× g for 20 min at 4 °C to separate the Au@Pt NP conjugate. The synthesized Au@Pt NP conjugate was resuspended in conjugate buffer. This Au@Pt–streptavidin conjugate was conjugated with 100 nM of biotin-labeled polyA-80 ssDNA reporter and shaken (45-50 rounds/min) for 30 min at 37 °C. Unbound ssDNA were removed through centrifugation at 10,000× g for 20 min at 4 °C and further stored at 4 °C.

The Au NPs and Au@Pt NPs were characterized with TEM using a JEM CX-100 transmission electron microscope (Jeol, Tokyo, Japan), as described in Section 4.1. Typical TEM images for AMPs and SMP are presented in Figure S3. The Au NPs, Au@Pt NPs, Au@Pt–streptavidin conjugate, and Au@Pt–streptavidin–polyA-80 conjugate were characterized with DLS using a Zetasizer Nano ZSP (Malvern Panalytical, Malvern, UK), as described in Section 4.2. Results are presented in Figure S4.

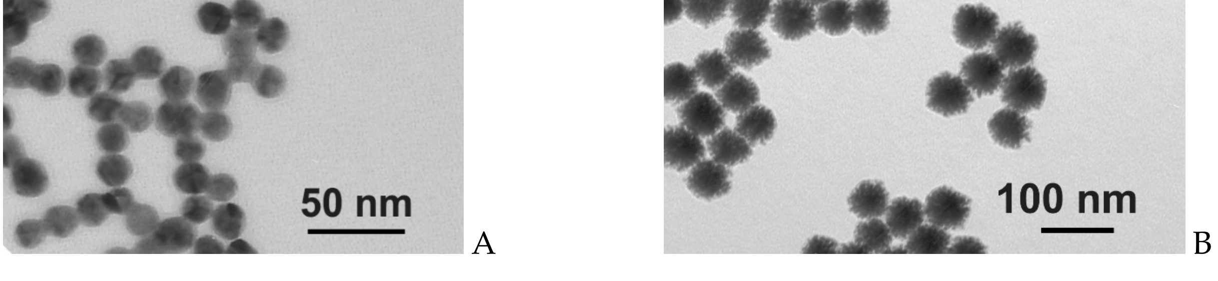

Figure S3. TEM images of Au NPs **(A)** and Au@Pt NPs **(B).**

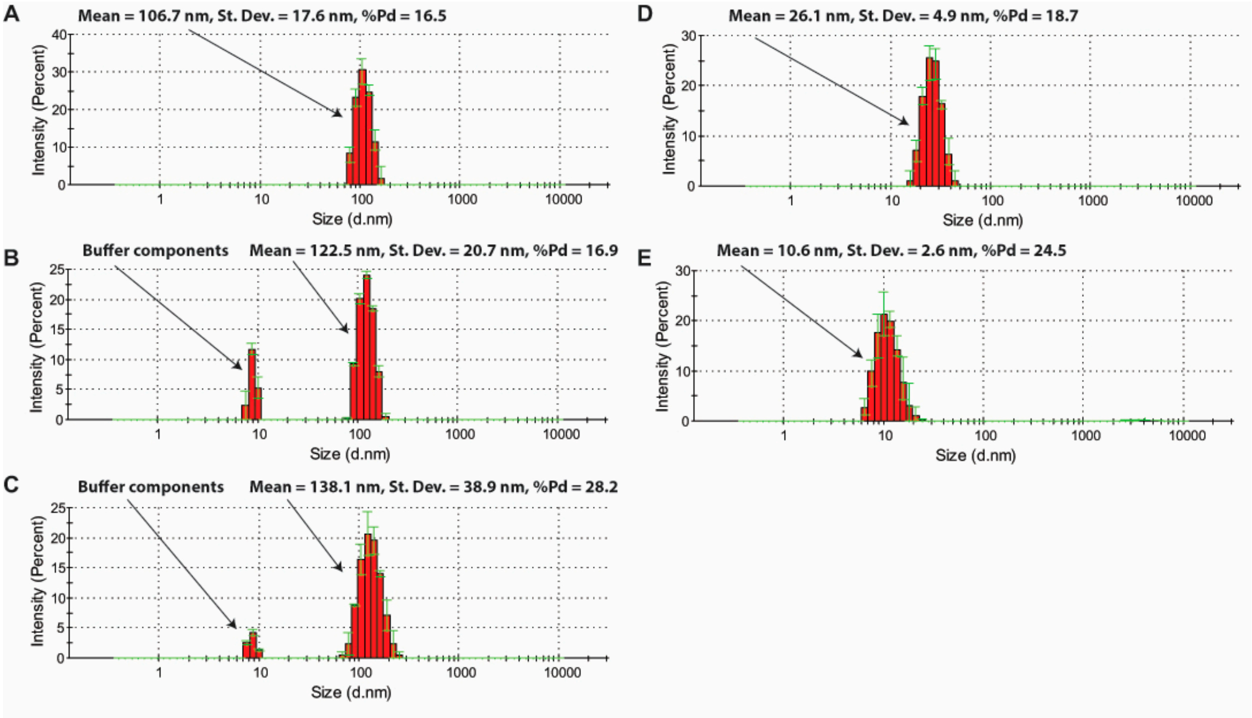

Figure S4. Characterization of Au NPs, Au@Pt NPs, Au@Pt–streptavidin conjugate, and Au@Pt–streptavidin–polyA-80 conjugate using DLS. Distributions of hydrodynamic diameters of Au@Pt NPs **(A)**, Au@Pt–streptavidin conjugate **(B)**, Au@Pt–streptavidin–polyA-80 conjugate **(C)**, Au NPs **(D)**, and buffer for conjugate storage **(E)**. Mean—mean hydrodynamic diameters, St. Dev.—standard deviation, %Pd—polydispersity.

Section S6. Trans-cleavage of immobilized ssDNA

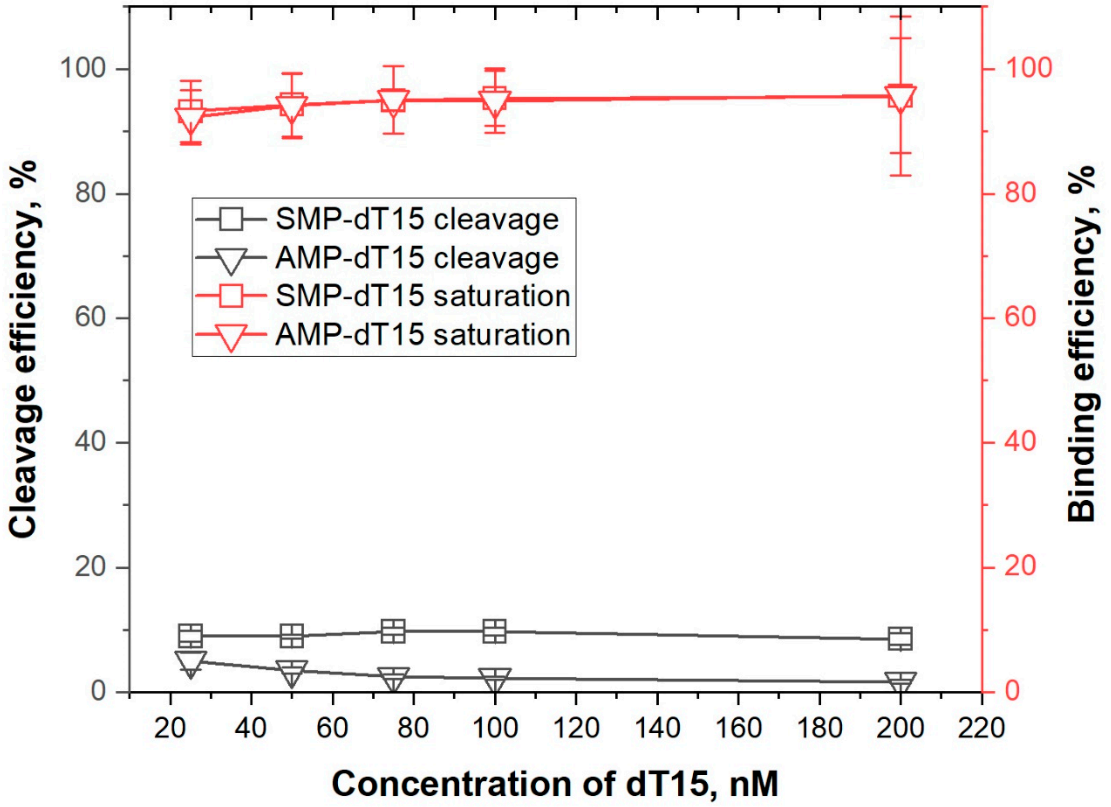

Figure S5. Binding and cleavage of poly-dT-15 upon different concentrations of the targets and constant concentration of MPs (0.0625%).

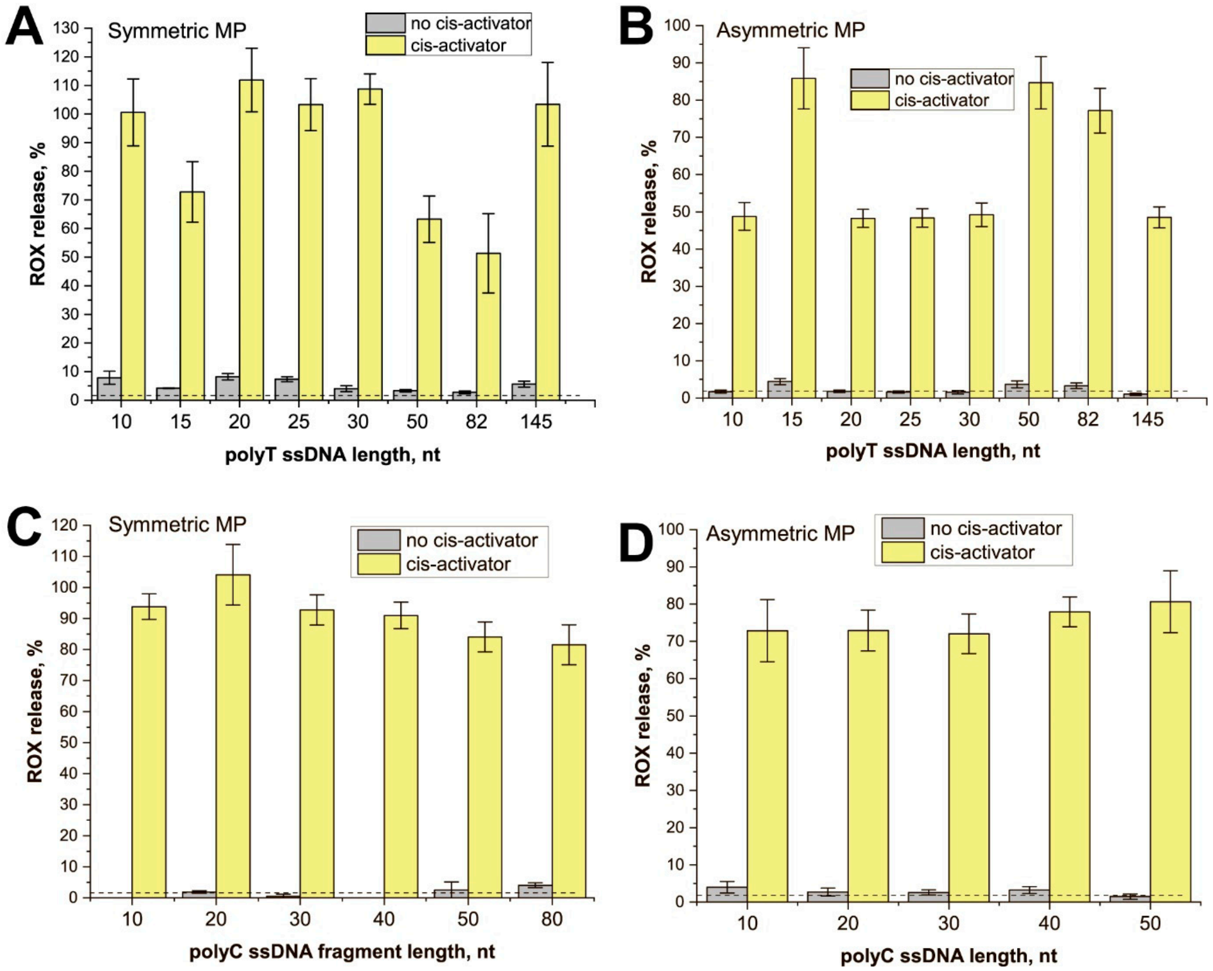

Figure S6. Control measurements of ROX–15dT–BHQ2 probe cleavage. (**A**) SMP–polyT conjugates, (**B**) AMP–polyT conjugates, (**C**) SMP–polyC conjugates, and (**D**) AMP–polyC conjugates.

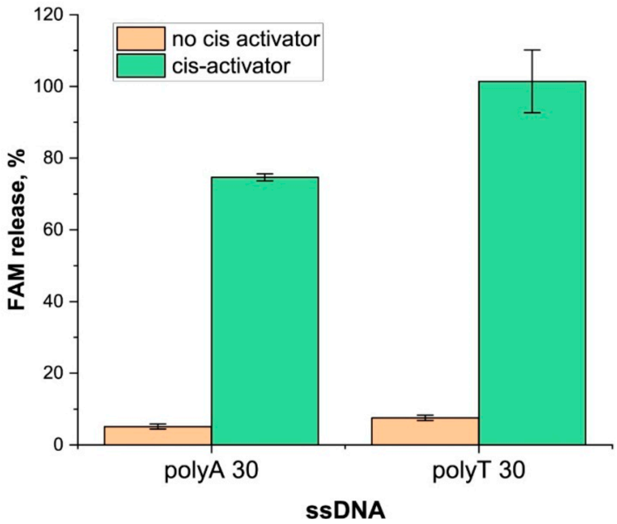

Figure S7. Control measurements of polyA and polyT probe cleavage with following conjugation.

Section S7. Attached ssDNA oligonucleotides that were used in previous research

Table S3. ssDNA oligonucleotides used in previous research.

Surface type	Type of ssDNA probe	Length of ssDNA, nt	Ref.
Plain gold	Poly T	10	[17]
	PolyA	20	
	polyA	30	
Plain gold	Hairpin		[21]
Plain polystyrol	ds-ssDNA(polyT) composite	15	[23]
Plain gold	ds-ssDNA(A-T mix) composite	10	[24]
Plain gold	Hairpin, T-rich loop	15	[40]
	polyT	20	
Plain platinum	A-T-G-C mix	20	[20]
Plain gold–GNP	A-T-G-C mix	18	[39]
MP	polyA	45	[19]
GNP–GNP	ds-ssDNA(polyA) composite	10	[22]

Section S8. Detection of dsDNA targets using CRISPR–Cas12 assay in homogeneous format

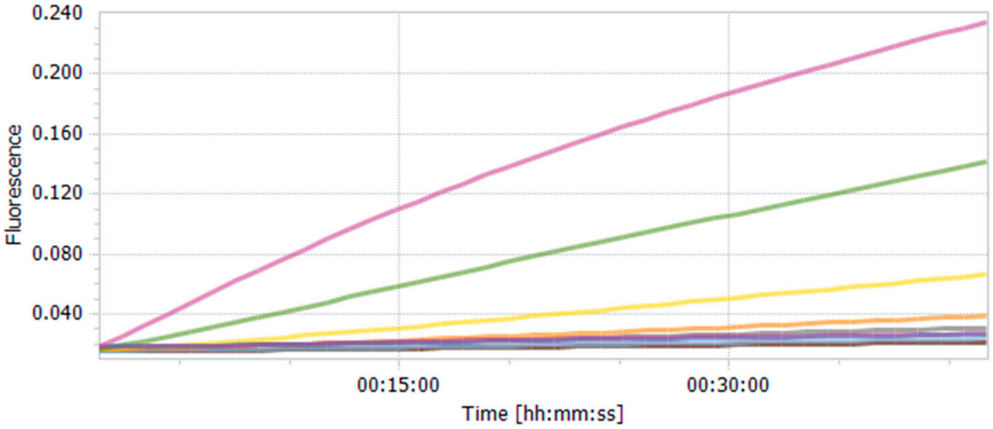

A

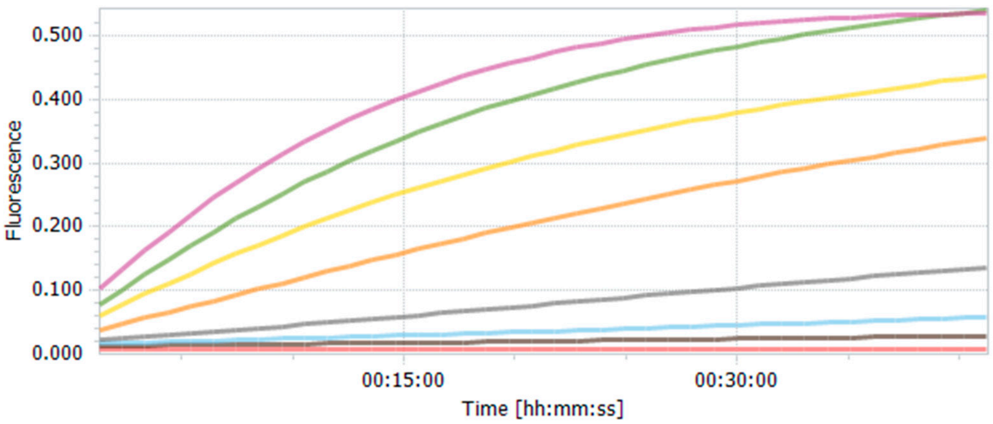

B.

Figure S8. Fluorescence plots of a cleaved ROX–dT15–BHQ2 probe in results of detection of dsDNA targets using CRISPR–Cas12 assay in homogeneous format. **(A)** dsDNA target of *D. solani* at different concentrations (the fluorescent signal decreases sequentially in the series 1000, 330, 110, 37, 12, 4, and 0 pM). **(B)** dsDNA target of *E. amylovora* at different concentrations (the fluorescent signal decreases sequentially in the series 10000, 3330, 1110, 370, 120, 41, 13, and 0 pM).

Section S9. Characteristic of Au@Pt–streptavidin–polyA-80 conjugate

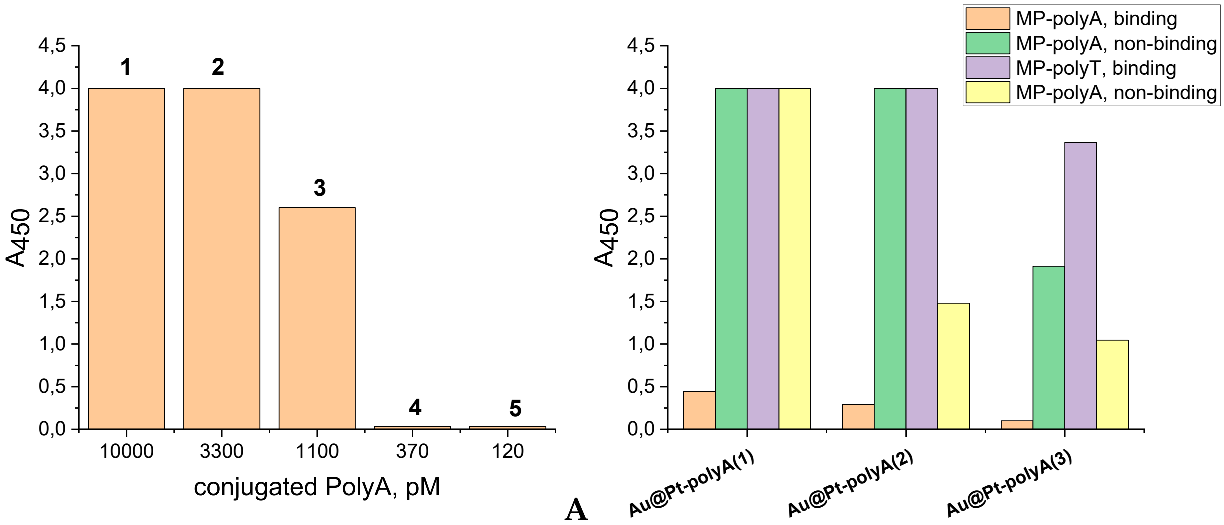

A B

Figure S9. Estimation of different dilutions of Au@Pt–streptavidin–polyA-80 conjugate in the reaction with TMB-based substrate solution for peroxidase (numbers indicate conjugate dilutions containing corresponding amounts of polyA-80, 1 – 10000, 2 – 3300, 3 – 1100, 4 – 370, and 5 – 120 pM). **(A)** Before interaction with MP–ssDNA conjugate. **(B)** After interaction with MP–polyA-80 and MP–polyT-80 conjugates.

We analyzed the predicted Kd for the interaction of polyT and polyA with different lengths based on different NN-models using the MeltingTemp.py module from the biopython project [45].

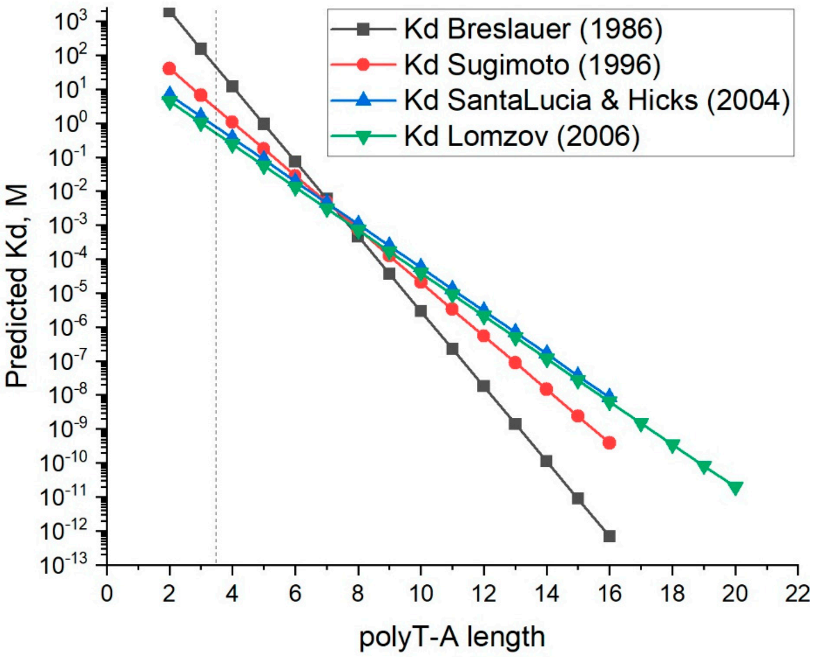

Figure S10. Dependences of predicted Kd on polyT–A length based on different NN-models. The dash line separates the calculated values for DNA length out of verified models (<4 nt) and values for DNA lengths within experimentally verified
